# An efficient protocol for total DNA extraction from the members of order Zingiberales- suitable for diverse PCR based downstream applications

**DOI:** 10.1186/2193-1801-2-669

**Published:** 2013-12-13

**Authors:** Khumallambam Devala Devi, Kshetrimayum Punyarani, Nandeibam Samarjit Singh, Huidrom Sunitibala Devi

**Affiliations:** Medicinal Plants & Horticultural Resources Division, Institute of Bioresources and Sustainable Development, Takyelpat Institutional Area, Imphal, 795001 Manipur India

**Keywords:** CTAB, DNA extraction, DNA barcoding gene, ISSR, RAPD, Virus detection, Zingiberales

## Abstract

Different protocols are usually used for extracting total deoxyribonucleic acid (DNA) from different plant species of same order and DNA of the associated viruses. Here, we describe a rapid, efficient and universal protocol for isolating total DNA from the members of Zingiberales which harbor a high amount of polysaccharides and secondary metabolites. DNA isolated with this protocol was successfully used for PCR based downstream applications viz. random amplified polymorphic DNA (RAPD), Inter-simple sequence repeats (ISSR), DNA barcoding gene (Internal transcribed spacer and trnl-f) amplification and detection of the viruses.

## Introduction

Molecular techniques require isolation of genomic DNA of suitable purity. The isolation of good quality DNA is the prerequisite for molecular research. Successful application of PCR based downstream applications requires efficient recovery of good quality and quantity of DNA. To isolate pure and intact DNA from plant tissues, numerous protocols have been established (Saghai-Maroof et al. 1984; Doyle and Doyle 1990; Scott and Playford 1996; Sharma et al. 2000; Pirttilä et al. 2001; Drábková et al. 2002; Shepherd et al. 2002; Mogg and Bond 2003; Haymes 1996). However, plant species belonging to the same or related genera can exhibit enormous variability in the complexity of pathways of dispensable functions. Thus, these DNA extraction protocols cannot be reproduced for all plant species (Porebski et al. 1997; Ribeiro and Lovato 2007). The cetyl trimethylammonium bromide (CTAB) method and its modifications have been used to obtain good quality total DNA for polymerase chain reaction (PCR) based downstream applications. Various commercial extraction kits such as DNeasy Plant Mini kits (Qiagen Valencia, CA, USA) are available, but the main problem with these commercially available kits is their high cost per sample (Ahmed et al. 2009). The use of multiple extraction protocols is laborious, time consuming and expensive. These necessitate the development of a universal protocol for isolating DNA from different plant species. In many cases, it has been observed that different methods are usually used for extraction of plant genomic DNA (Doyle and Doyle 1990; Porebski et al. 1997) and DNA of viruses (Thomas and Dietzgen 1991; Xei and Hu 1995; Ahlawat et al. 1996) associated with the plant. The members of the order Zingiberales contain a high amount of polysaccharides and polyphenols. Secondary metabolites and polysaccharides interfere with total DNA isolation procedures and PCR based downstream applications. The removal of such contaminants needs complicated and time-consuming protocols. Here, in the present study, we describe a simple, rapid, reliable and inexpensive CTAB based method for the extraction of high quality total DNA of different species of Zingiberales and associated viruses. The isolated high quality genomic DNA is amenable to RAPD (Random amplified Polymorphic DNA), ISSR (Inter-simple sequence repeats), amplification of plant barcode genes (ITS and trnL-F) and detection of virus associated with *Musa* with reduced cost and health concerns.

## Material and methods

### Plant material

Fresh, young and tender leaves of five different species of the order Zingiberales (*Kaempferia galanga*, *Kaempferia marginata*, *Zingiber officinale*, *Zingiber zerumbet*, *Musa* sp.) which contain high amounts of polysaccharides and polyphenols were collected and wiped with 70% ethanol. For PCR based detection of virus, leaves of banana plant infected with two viruses associated with *Musa* sp. i.e. Banana bunchy top virus (BBTV) and Banana streak virus (BSV) were collected. A minimum of ten replicates were taken for each species.

### Reagents

- 100 mM Tris–HCl (pH 8),
- 1.4 M Sodium Chloride (NaCl),
- 20 mM-Ethylenediaminetetraacetic acid (EDTA) (pH 8),
- 2% (w/v) Cetyl trimethylammonium bromide (CTAB)
- Chloroform-isoamyl alcohol (24:1)
- Isopropanol, 70% ethanol
- TE buffer (pH 8): 10 mM Tris–HCl, 1 mM EDTA
- 0.5× Tris/Borate/EDTA (TBE) (10× stock contained 1 M Tris, 0.8 M boric acid, 0.5 M EDTA)
- Agarose (molecular grade)

*

### DNA extraction protocol

1. Preheat the extraction buffer containing 100 mM Tris–HCl (pH 8), 1.4 M NaCl, 20 mM
  EDTA (pH 8), 2% (w/v) CTAB in water bath at 60°C for about 15 minutes.
2. Submerge 1 g of plant tissue in 5 ml of absolute alcohol for 5 minutes and allow alcohol to evaporate.
3. Grind the tissue in presence of 1% PVP (Polyvinylpyrrolidone) and pre-warmed extraction buffer by using a pre-chilled mortar and pestle (-40°C/-80°C) at room temperature.
4. Transfer the ground material into 2 ml centrifuge tubes and incubate in water bath at 60°C for 1 hour.
5. Centrifuge the tubes at 10,000 rpm for 10 minutes at 4°C and collect the supernatant in 1.5 ml centrifuge tube using wide bored tip.
6. To the supernatant add equal volume of chloroform: isoamyl alcohol (24:1) and mix by inversion for 15 minutes.
7. Centrifuge the tubes at 10,000 rpm for 10 minutes at 4°C and collect the supernatant in 1.5 ml centrifuge tube.
8. Again add equal volume of chloroform: isoamyl alcohol (24:1) to the supernatant and mix by inversion for 15 minutes.
9. Centrifuge the tubes at 10,000 rpm for 10 minutes at 4°C and collect the supernatant.
10. To the supernatant add twice the volume of chilled isopropanol to precipitate the DNA and incubate it at -20°C for 30 minutes.
11. Centrifuge the tubes at 10,000 rpm for 10 minutes at 4°C and collect the pellet.
12. Wash the pellet 2–3 times with 70% ethanol and air dry the pellet in room temperature.
13. Add 50–100 μl of TE buffer to dissolve the DNA.
14. Store at -20°C for further use.

### Quantification and visualization of DNA

DNA was quantified by measuring optical density (O.D.) at A260 and A280 with a *Nanodrop Spectrophotometer* (*ND2000*). Samples were subjected to electrophoresis in 1× TBE buffer for 1 hour at 80 V. 5 μl of the isolated genomic DNA was loaded on 0.8% agarose gel stained with ethidium bromide to check DNA quality. The gels were photographed under a *Gel Documentation system* (*Perkin Elmer Geliance 200*).

### RAPD and ISSR study

PCR amplification of ten replicates of genomic DNA of *Zingiber zerumbet* and *Zingiber officinale* was carried out using RAPD and ISSR primers respectively which were synthesized by *Sigma Aldrich Chemicals Pvt. Ltd.*, *India* as per the sequence of *Operon Technologies*, *Inc.*, *USA*. PCR amplifications were performed routinely using the following PCR reaction mixture: 25 μl contained 50 ng of template DNA, 1× PCR buffer, 1.5 mM of magnesium chloride (MgCl_2_) 200 μM of deoxynucleotide triphosphates (dNTPs), 10 picomol of each primer, and 1 U of *Taq* polymerase. PCR amplification was carried out in a thermal Cycler (*Eppendorf Mastercycler Pro S*). Thermal cycling conditions were as follows: initial denaturation step for 5 min at 94°C, followed by 35 cycles each of 1 min at 94°C (denaturation), 1 min at 37°C (annealing), 2 min at 72°C (extension) followed by one final extension of 7 min at 72°C. The annealing temperatures of ISSR primers were different for each primer depending upon the melting temperature. The amplification products were electrophoresed in 1.8% agarose gels in 0.5× TBE (10× stock contained 1 M Tris, 0.8 M boric acid, 0.5 M EDTA) and stained with ethidium bromide (0.5 μg/ml). The gels were photographed under a *Gel Documentation system* (*Perkin Elmer Geliance 200*).

### ITS and trnL-F gene amplification

Genomic DNA of *Kaempferia galanga* and *Kaempferia marginata* isolated by the present method was used as template for nuclear and chloroplast gene amplification. Ten replicates of *Kaempferia galanga* DNA was amplified with primers ITS1 (5′-TCCGATGGTGAACCTGCGG-3′) and ITS4 (5′-TCCTCCGCTTATTGATATGC-3′). Ten replicates of using *Kaempferia marginata* DNA was amplified with primers trnL-Fc (5′-GAAATCGGTAGACGCTACG-3′) and trnL-Ff (5′-ATTTGAACTGGTGACACGAG-3′). The PCR reaction mixture composition was same as that used for RAPD analysis. The reaction mixture of 25 μl contained 50 ng of template DNA, 1× PCR buffer, 1.5 mM of MgCl_2_, 200 μM of dNTPs, 0.25 μM of each primer, and 1 U of *Taq* polymerase. PCR amplification was carried out in a thermal Cycler (*Eppendorf Mastercycler pro S*). Thermal cycling conditions were as follows: initial denaturation step for 5 min at 94°C, followed by 35 cycles each of 1 min at 94°C (denaturation), 1 min at 59.3°C for ITS1 and ITS4 and 61.8°C for trnL-Fc and trnL-Ff (annealing), 2 min at 72°C (extension) followed by one final extension of 7 min at 72°C. The amplification products were electrophoresed in 1.8% agarose gels in 0.5× TBE (10× stock contained 1 M Tris, 0.8 M boric acid, 0.5 M EDTA) and stained with ethidium bromide (0.5 μg/ml). The gels were photographed under a *Gel Documentation system* (*Perkin Elmer Geliance 200*).

### Virus DNA detection

BBTV and BSV were detected by PCR using specific primers of BBTV: BTVCPF (forward primer) 5′- GCTAGGTATCCGAAGAAATC-3′, BTVCPR (reverse primer) 5′- TCAAACATGATATGTAATTC-3′ (Burns et al. 1995) and BSV: Mys-F1 5′- TAAAAGCACAGCTCAGAACAAACC-3′, Mys R1 5′-CTCCGTGATTTCTTCGTGGTC-3′ (Geering et al. 2000). Virus DNA amplifications were performed routinely using PCR reaction mixture of 25 μl containing 100 ng of template DNA, 1× PCR buffer, 1.5 m MgCl_2_, 0.2 mM of each dNTPs, 50 ng forward primer, 50 ng reverse primer and 1 U of *Taq* polymerase. PCR amplification was carried out in a thermal cycler (*Eppendorf Mastercycler Pro S*). Thermal cycling conditions were as follows: BBTVCPF- 94°C for 3 min, then subjected to 35 cycles of 94°C for 45 s, 50°C for 45 s, and 72°C for 1 min; and finally 1 cycle of 72°C for 10 min and for BSV Mys F1and R1- 94° 4 min, 50° 1 min; 72° 2 min for 1 cycle, then subjected to 30 cycles 94° 1 min; 50° 1 min; 72° 2 min and finally 1 cycle of 72° 10 min. Amplification products were electrophoresed in 1.8% agarose gels in 0.5× TBE (10× stock contained 1 M Tris, 0.8 M boric acid, 0.5 M EDTA) and stained with ethidium bromide(0.5 μg/ml). The gels were photographed under a *Gel Documentation system* (*Perkin Elmer Geliance 200*).

## Results and discussion

**Figure 1:**
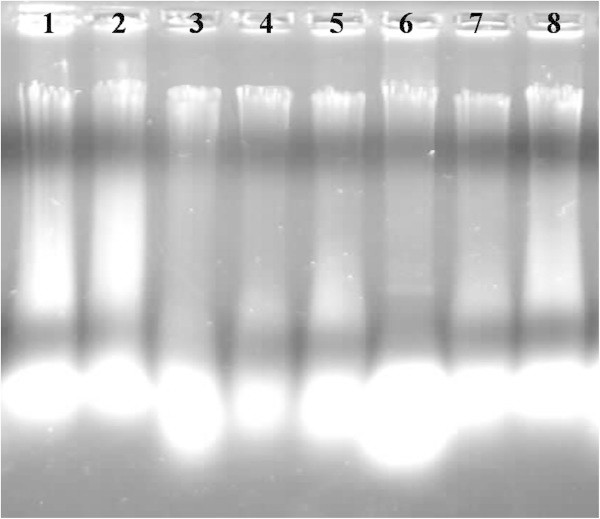
**Electrophoresis of total DNA extracted from different species of Zingiberales using CTAB method, lane 1–2: Genomic DNA of** ***Kaempferia marginata*** **, 3–4: Genomic DNA of** ***Zingiber officinale*** **, 5–6: Genomic DNA of** ***Zingiber zerumbet*** **, 7–8: Genomic DNA of** ***Musa*** **sp. in 0.8% agarose.**

**Figure 2:**
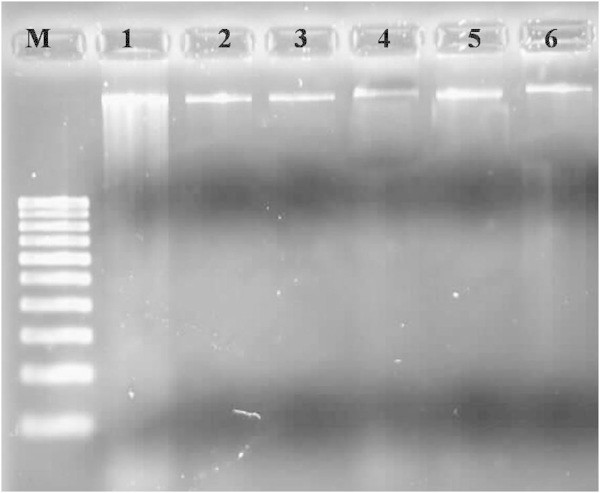
**Electrophoresis of total DNA extracted from different species of Zingiberales, lane 1- Genomic DNA of** ***Kaempferia galanga*** **isolated using CTAB method, 2- Genomic DNA of** ***Kaempferia galanga*** **using our method, 3- Genomic DNA of** ***Kaempferia marginata*** **using our method, 4- Genomic DNA of** ***Zingiber officinale*** **using our method, 5- Genomic DNA of** ***Zingiber zerumbet*** **using our method, 6- Genomic DNA of** ***Musa*** **sp. using our method in 0.8% agarose.**

**Figure 3:**
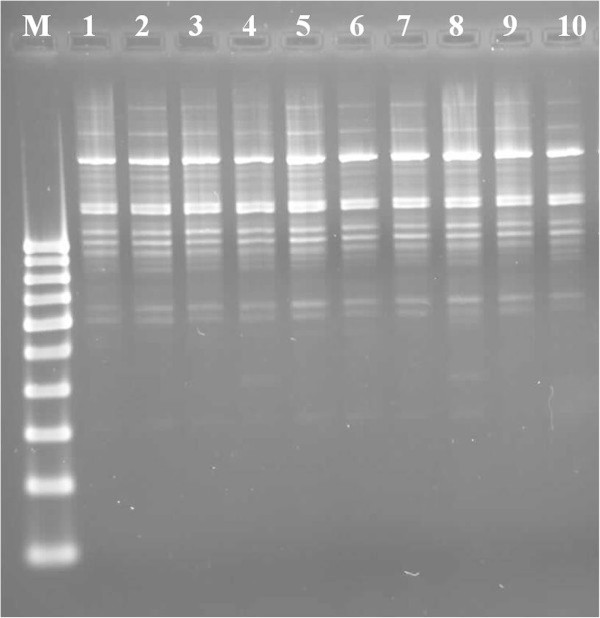
**RAPD profile of** ***Zingiber zerumbet*** **using the DNA extracted by our method as template DNA with OPC-09 (M-100 bp DNA ladder; 1 to10- replicates of** ***Zingiber zerumbet*** **).**

**Figure 4:**
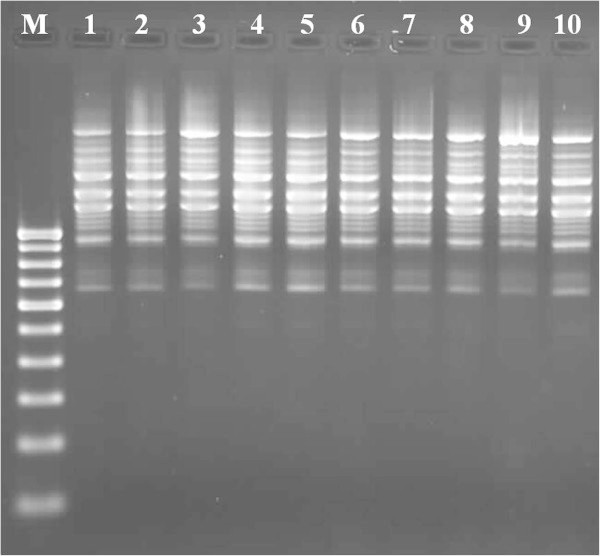
**ISSR profile of** ***Zingiber officinale*** **using the DNA extracted by our method as template DNA with ISSR-6 (M-100 bp DNA ladder; 1 to10- replicates of** ***Zingiber officinale*** **).**

**Figure 5:**
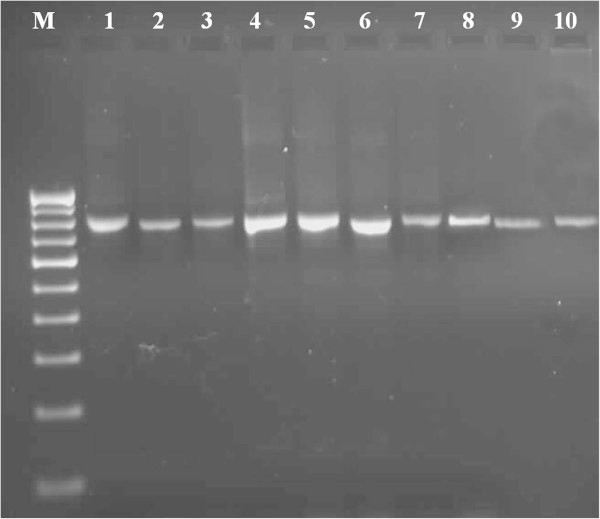
**Amplification of ITS gene of** ***Kaempferia galanga*** **using the DNA extracted by our method as template DNA (M-100 bp DNA ladder; 1 to10- replicates of** ***Kaempferia galanga*** **).**

**Figure 6:**
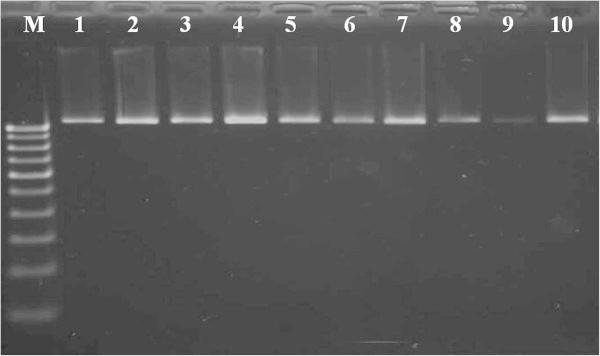
**Amplification of trnlf gene of** ***Kaempferia marginata*** **using the DNA extracted by our method as template DNA (M-100 bp DNA ladder; 1 to10- replicates of** ***Kaempferia marginata*** **).**

**Figure 7:**
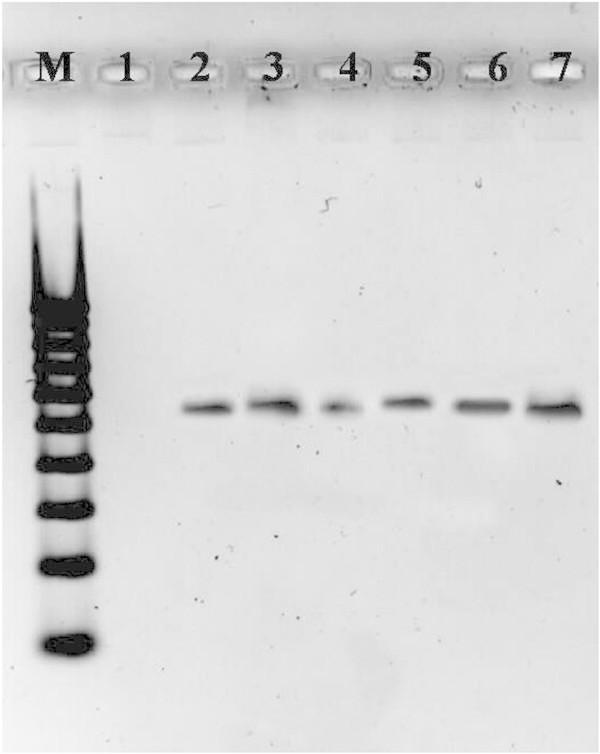
**PCR based detection of BBTV using the DNA extracted by our method as template DNA (M-100 bp DNA ladder; 1-Uninfected** ***Musa*** **sp. used as control; 2 to10- replicates of infected** ***Musa*** **sp. with BBTV).**

**Figure 8:**
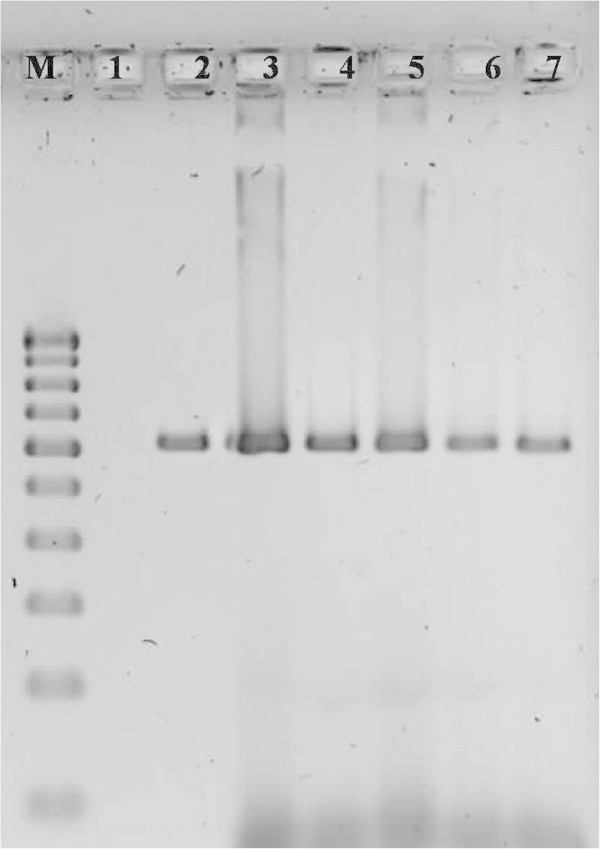
**PCR based detection of BSV using the DNA extracted by our method as template DNA (M-100 bp DNA ladder; 1-Uninfected** ***Musa*** **sp. used as control; 2 to10- replicates of infected** ***Musa*** **sp. with BSV).**

In this study, DNA quality was assayed by gel electrophoresis and intense bands were seen on 0.8% agarose. Genomic DNA isolated using CTAB method (Doyle and Doyle 1990) did not produce distinct and intact band (Figures 1 and 2 lane-1) and presence of smear which hinders PCR downstream applications for producing scorable bands. However, total genomic DNA prepared with our protocol showed no degradation and smear (Figure 2 lane 2–6). The success of the optimized extraction method in obtaining high-quality genomic DNA from all the tested Zingiberales demonstrated the broad applicability of the method. Although, better yield was obtained in case of *Kaempferia galanga* using traditional method, the quality of the band was not good (Table 1). However, in other species better yield and quality was obtained using our method (Table 1). The average yield of total nucleic acid from 1 g of plant material using our method ranged from 879.59 μg/μl to 2350.81 μg/μl which is much higher than those obtained with kits and normal CTAB method. The highest concentration of DNA was obtained in *Kaempferia galanga* (Table 1). Tukey’s comparison test showed the average yield of *K. galanga* was significantly higher than other species (Table 1). The A260/A280 ratio was in the range of 1.82 to 1.94 which indicated the purity of the nucleic acid obtained using our method and insignificant/low levels of proteins and polysaccharide contamination. Although, there was significant difference in the concentration of DNA, variance analysis showed that there was no significant difference in purity of the nucleic acid obtained using our method. Previous reports on high-quality plant DNA extraction methods (Aljanabi and Martinez 1997; Zhang and Steward 2000; Karakousis and Langridge 2003; Manen et al. 2005; Bokszczanin and Przybyla 2006; Chakraborti et al. 2006; Arbi et al. 2009; Biswas and Biswas 2011; Japelaghi et al. 2011) used liquid nitrogen, lyophilization (freeze-drying), alternating cold (about -80°C), enzymatic digestion for grinding and/or rupturing of the cell and nuclear walls. However, recently, a modified DNA extraction protocol which neither utilizes liquid nitrogen, lyophilization (freeze-drying), alternating cold (about -80°C), nor enzymatic digestion for grinding and/or rupturing of the cell and nuclear walls, has been reported (Agbagwa et al. 2012). Using their method, one person is able to process as many as 200 samples in a 5-day working period with a labor cost as low as 100 to US$110 or between 1.8 and US$2 per leaf sample. However in the method reported here, unlike that reported above (Agbagwa et al. 2012), no hazardous chemicals such as β-mercaptoethanol, phenol and Rnase were used. In comparison with recently reported method (Agbagwa et al. 2012) which took 220 minutes approximately, this method eliminates much of the time consuming steps allowing the whole procedure to be completed within 160 minutes. High purity DNA is required for PCR and other PCR-based techniques, such as random amplified polymorphic DNA (RAPD), micro- and macrosatellite analyses, restriction fragment length polymorphism (RFLP) and amplified fragment length polymorphism (AFLP) used for genome mapping and DNA fingerprinting (Khanuja et al. 1999). The DNA extracted by this method yielded reproducible and scorable bands proving its suitability for PCR applications using RAPD (Figure 3), ISSR (Figure 4), nuclear and chloroplast gene marker for diverse molecular studies (Figures 5 and 6) and also for detection of viruses (Figures 7 and 8). This protocol involves less steps, and time therefore is more economical (as cost per sample is reduced) when compared to traditional techniques (Nickrent 1994) (Triboush et al. 1998). The efficiency and the speed of this method together with the use of inexpensive facilities and the absence of toxic chemicals make the present method a noticeable alternative for the extraction of high quality and yield of nucleic acid from most plant species, especially from members of the order Zingiberales. The modified CTAB method described in this paper has already been adopted for routine use in our laboratory. This method is widely applicable to extract total DNA from the members of order Zingiberales and its associated viruses. Furthermore, since, less steps and time are involved, this method is more economical. Hence, our protocol is more suitable for developing countries in Africa and Asia.

**Table 1:** **Quantitative estimates of DNA concentration revealed by Nanodrop Spectrophotometer**

Species	Concentration of DNA obtained using CTAB method μg/μl*	Concentration of DNA obtained using our method μg/μl*	A_260_/A_280_* (CTAB method)	A_260_/A_280_* (our method)
*Kaempferia galanga*	2456.20^a^	2350.81^a^	3.32^b^	1.82^a^
*Kaempferia marginata*	56.26^b^	879.59^b^	2.61^a^	1.94^a^
*Zingiber officinale*	31.23^c^	1287.75^b^	2.62^a^	1.94^a^
*Zingiber zerumbet*	99.20^d^	1305.59^b^	3.73^b^	1.85^a^
*Musa sp.*	73.83^e^	1065.97^b^	2.64^a^	1.93^a^

*Means of 10 samples.

*Means followed by same letters are not significantly different at p < 0.05, according to Tukey’s comparison test.

## Abbreviation

AFLP: Amplified fragment length polymorphism
BBTV: Banana bunchy top virus
BSV: Banana streak virus
CTAB: Cetyl trimethylammonium bromide
dNTPs: Deoxynucleotide triphosphates
EDTA: Ethylene diaminetetraaceticacid
HCl: Hydrochloric acid
ISSR: Inter-simple sequence repeats
ITS: Internal transcribed spacer
MgCl2: Magnesim chloride
PCR: Polymerase chain reaction
RAPD: Random amplified polymorphic DNA
RFLP: Restriction fragment length polymorphism
TBE: Tris/borate/EDTA
TE: Tris EDTA.
